# Systems Pharmacology of the NGF Signaling Through p75 and TrkA Receptors

**DOI:** 10.1038/psp.2014.48

**Published:** 2014-12-03

**Authors:** T Toni, P Dua, P H van der Graaf

**Affiliations:** 1 Department of Life Sciences, Imperial College London, London, UK; 2 Pharmatherapeutics Research Clinical Pharmacology, Pfizer Neusentis, Cambridge, UK; 3 Leiden Academic Centre for Drug Research (LACDR), Systems Pharmacology Cluster, Leiden, The Netherlands

## Abstract

The nerve growth factor (NGF) pathway has been shown to play a key role in pain treatment. Recently, a systems pharmacology model has been proposed that can aid in the identification and validation of drug targets in the NGF pathway. However, this model did not include the role of the p75 receptor, which modulates the signaling of NGF through the tropomyosin receptor kinase A (TrkA). The precise mechanism of the interaction between these two receptors has not been completely elucidated, and we therefore adopted a systems pharmacology modeling approach to gain understanding of the effect of p75 on the dynamics of NGF signal transduction. Specifically, models were developed for the so-called heterodimer and for the ligand-passing hypotheses. We used the model to compare the effect of inhibition of NGF and TrkA and its implication for drug discovery and development for pain treatment.

Nerve growth factor (NGF) signaling plays a key role in neuronal development, function, survival, and growth.^1^ NGF signals by binding two types of membrane receptors: tropomyosin-related kinase A (TrkA) and p75 neurotrophin receptor (p75), a member of the tumor necrosis factor superfamily.^1,2^ Both receptors can function independently by interacting with NGF, each with a relatively low affinity^1,2,3^ (**Figure 1a**). However, when both receptors are coexpressed, they produce so-called high-affinity binding sites for NGF, which alter the signaling.^2,4^ It has been shown that p75 increases NGF binding affinity and specificity to TrkA receptor.^5,6^ Moreover, in presence of p75 receptors, lower NGF concentrations are needed to elicit TrkA-dependent responses in neurons.^7,8^ However, the precise mechanism by which p75 and TrkA receptors interact has not been known and has occupied scientist for more than two decades.^9^

Two different hypotheses for the p75–TrkA interaction mechanism have been proposed and studied experimentally and computationally.^3,10^ In the ligand-passing mechanism (**Figure 1c**–**e**), p75 receptor rapidly binds NGF, increasing its local concentration, and then passes NGF to the TrkA receptor. This hypothesis does not require a direct interaction between p75 and TrkA. The ligand-passing hypothesis has been supported by a number of studies including ligand mutagenesis^11^ and ligand blocking antibodies to p75,^12^ both of which result in reduced TrkA activation. In the heterodimer mechanism (**Figure 1b**), p75 and TrkA receptors are physically associated by forming a heterodimer that is thought to increase affinity of the NGF binding to TrkA, possibly through a conformational change in TrkA. In this mechanism, p75 and TrkA interact though their cytoplasmic and transmembrane domains and form complexes even prior to NGF stimulation.^3,13^ The evidence for the heterodimer hypothesis has been provided by coimmunoprecipitation studies which documented p75–TrkA complexes.^14,15^ The heteroreceptor was only observed in the absence of NGF. In the presence of NGF, however, the complex of p75 and TrkA is transient and quickly dissociates, leaving NGF bound to TrkA.^15^

Once NGF is bound to the TrkA receptor, it triggers TrkA receptor autophosphorylation. This in turn leads to recruitment of several adaptor proteins (including Grb2, Shc, and SOS) to the plasma membrane, which assists the activation of the MAPK phosphorylation cascade consisting of the Ras-Raf-Mek-Erk kinases. The NGF pathway and transduction of signal from the membrane to cell nucleus have been extensively and quantitatively studied.^16,17^

The NGF pathway has recently been linked to chronic pain in a number of studies.^18,19,20,21^ In particular, it has been shown that NGF levels substantially increase in chronic pain states, that administration of NGF elicits pain, and that NGF mutations can be found in patients with pain insensitivity. Chronic pain affects millions of people worldwide, making it one of the most prevalent modern health problems. Chronic pain can have substantial impact on patients’ quality of life through physical and social disability. A variety of agents are available for pain treatment, including opioids and nonsteroidal anti-inflammatory drugs; however, many patients remain refractory to these treatments. Thus, there is a need for the development of additional treatments with better efficacy and toleration profiles.

Since NGF has been linked to pain, NGF and other protein members of the NGF signaling pathway became potential new drug targets for treating chronic pain. Inhibitors of NGF have been developed in the form of monoclonal antibodies and have been shown to have analgesic effects for certain types of pain.^20,21,22,23,24^ To quantify and study the effects of the NGF and TrkA inhibitors on the signal transduction through TrkA pathway, Benson *et al.*^25^ developed a quantitative systems pharmacology model. The model combines two mechanistic systems biology models of Sasagawa *et al.*^16^ and Fujioka *et al.*^17^ and complements them with a pharmacokinetic component. A model combining mechanistic descriptions of biological pathways (e.g., some sort of mechanistic pharmacodynamic model) with a pharmacokinetic model component forms a so-called quantitative systems pharmacology model.

Systems pharmacology is the application of systems biology principles to the field of pharmacology.^26,27,28,29,30^ Its aim is to understand the actions of drugs by considering targets in the context of biological networks in which they exist. One of the systems pharmacology goals is to use modeling to help decrease the high rate of attrition happening in the late stages of drug discovery pipelines. One of the reasons for failures in drug discovery and development is the lack of understanding of complex biological mechanisms. Biological networks can be very nonlinear due to an interconnected web of various complex regulatory motifs such as feed-forward and feedback loops, cascades which may amplify or dampen signals, and are largely affected by cross talk. Due to this complexity, it is very challenging to understand—especially based on intuition alone—how a signal travels through a pathway, as well as to what degree the signal is dampened by various pathway inhibitors. Quantitative mathematical models of signaling pathways are needed for thorough understanding of signal flow.

The aim of our work is to develop a quantitative systems pharmacology model of the NGF signaling pathway and use it to guide drug discovery and development programs for pain treatment. In this article, we extend the existing NGF signaling pathway model by adding the p75 receptor and its interaction with the TrkA receptor. We use the model to study the effect of two specific pathway inhibitors, the NGF inhibitor and the TrkA inhibitor. We aim to address the following questions: What is the quantitative contribution of p75 receptor to the NGF signaling? How does understanding of p75–TrkA interaction mechanisms affect pain treatment; i.e., can we predict appropriate drug doses independent of understanding the underlying p75–TrkA interaction mechanism, or is dosing dependent on the mechanism? In this article, we do not take sides for or against any of the two p75–TrkA interaction hypotheses. We develop mathematical models for both mechanisms and study the implications of each mechanism on inhibition of the NGF signaling pathway.

## Results

### Mechanistic models of p75–TrkA interaction

Based on the information on p75–TrkA interaction found in the literature and summarized in the introduction, we developed mechanistic mathematical models for the ligand-passing and heterodimer mechanism hypotheses. The reactions are given below. Kinetic rates and initial conditions are specified in the **Supplementary Data**.

We begin with a list of common reactions to both models, independent of whether the actual mechanism is ligand-passing or heterodimer. NGF binds to both receptors, TrkA and p75, with relatively low affinity. The affinity for both receptors is similar; however, the kinetics of binding and unbinding of NGF to the p75 receptor is faster than that to the TrkA receptor^2,31^ (**Table 1**). NGF exists as a dimer and binds a dimeric TrkA receptor. However, the NGF dimer binds p75 in monomeric form only; the formation of the NGF–p75 complex induces a conformational change such that the second p75 monomer is not able to bind.^32,33^ Accordingly, species TrkA in our model represents a TrkA dimer, NGFa NGF dimer, while species p75 represents a monomer. Other reactions common to both models include production and degradation of NGF, TrkA, and p75 proteins, reversible binding of NGF to TrkA dimer, reversible binding of NGF to p75, and autophosphorylation of TrkA_NGF.

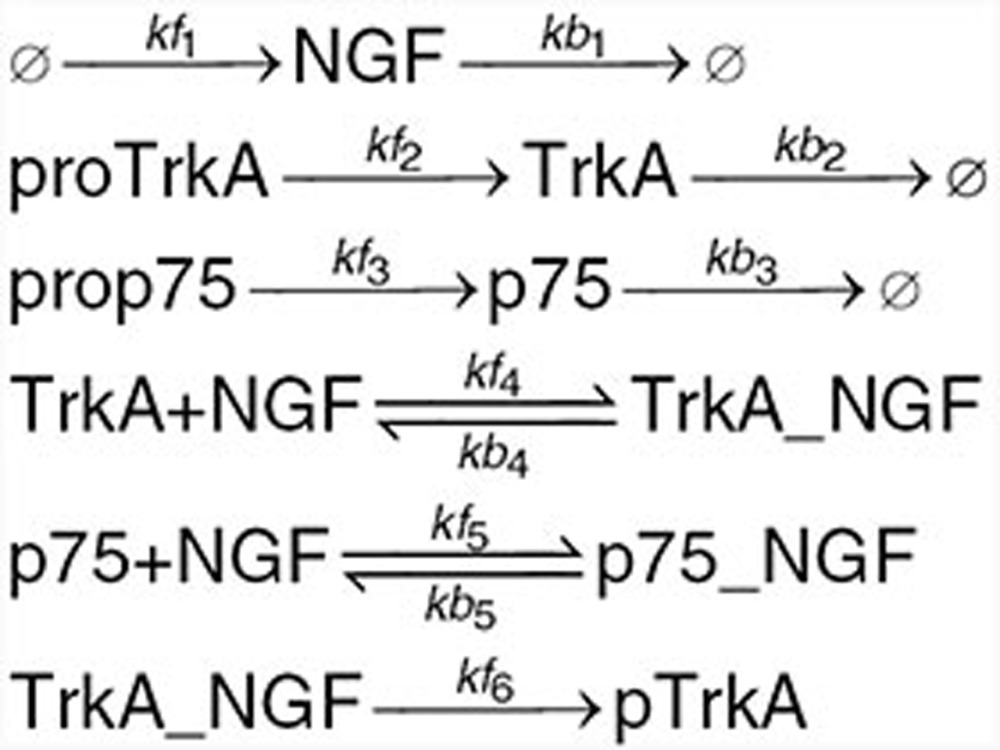


Under the heterodimer mechanism, TrkA and p75 form a heteroreceptor, which we represent by TrkA_p75_d_ in the model. The heteroreceptor can bind NGF, resulting in a complex TrkA_NGF_p75d. The rate of association increases 25-fold compared with binding of NGF to TrkA only and is commonly referred to “high-affinity binding”^31^ (**Table 1**). This complex is very short lived, with p75 rapidly dissociating and leaving TrkA dimer bound to the ligand NGF.

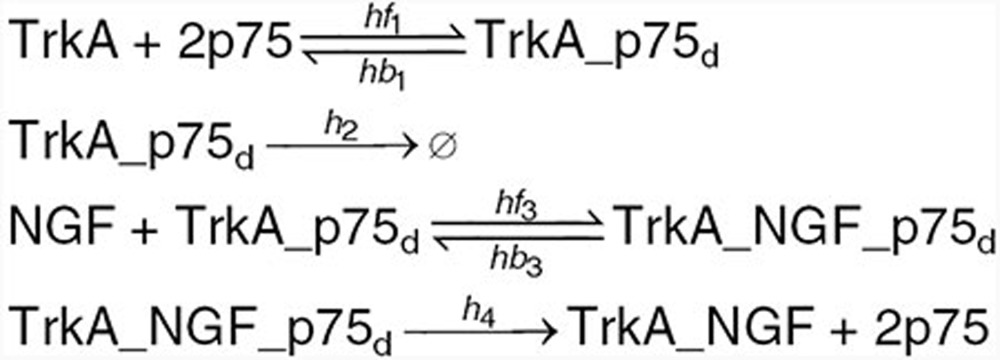


Under the ligand-passing mechanism, TrkA and p75 do not physically interact. NGF binds p75 monomer, which then forms a transient complex with TrkA dimer and finally dissociates into TrkA_NGF complex and a p75 monomer.

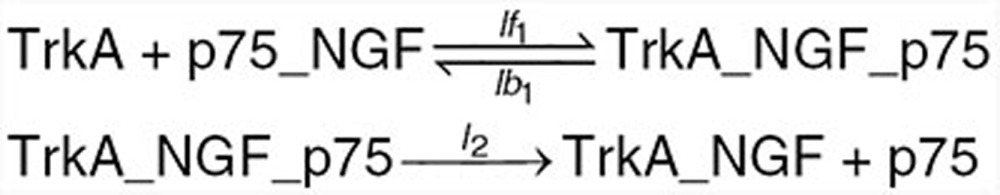


Benson *et al.* have translated this model into a two-compartmental cross-membrane model based on refs. 25,34. They have shown that incorporating physiological context into the model increased the accuracy of conclusions about the drug dose required to achieve pathway inhibition. The cross-membrane model reactions for the ligand-passing and heterodimer mechanisms, including parameter values and initial conditions, are provided in **Supplementary Data S2**. To develop models for a full NGF signaling pathway, we added the ligand-passing and heterodimer models described above to the NGF signaling pathway model proposed by Benson *et al.*,^25^ which is a combination of models by Sasagawa *et al.*^16^ and Fujioka *et al.*^17^

### Model simulation

**Figure 2:**
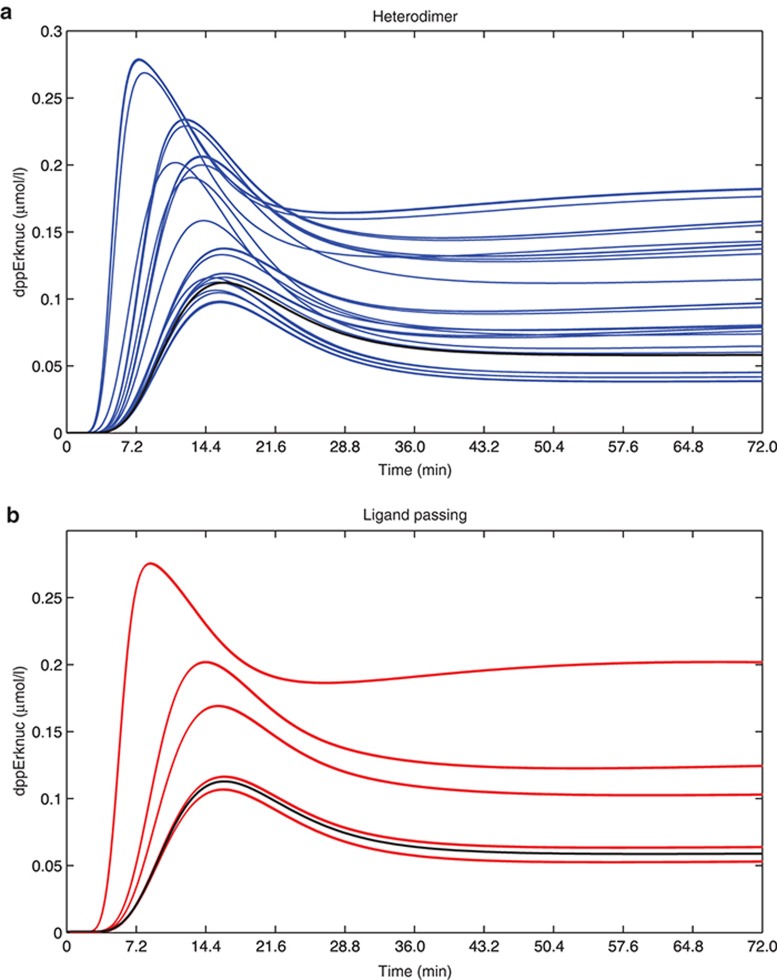
Simulation of models under NGF stimulation. Shown is the model output, the concentration time series of doubly phosphorylated nuclear Erk protein, denoted by dppErknuc. No inhibitors were present in the system (TrkAI = 0; NGFI = 0). (**a**) Simulation of the heterodimer model (blue lines). Parameters *k*_on_ and *k*_off_ were varied by taking all possible pairs of the following: *k*_off_ = 6.4 × {10^−5^, 10^−4^, 10^−3^, 10^−2^, 10^−1^, 1} s^−1^ and *k*_on_ = {0.005, 0.01, 0.05, 0.1, 1} M^−1^·s^−1^. (**b**) Simulation of the ligand-passing model (red lines). Parameters *k*_off_ and *k*_on_ were varied as in **a**. The black line in **a** and **b** shows the simulation of the model without the added p75 component; i.e., the black line corresponds to the simulation of the Sasagawa/Benson model, which was here simulated as the heterodimer (or ligand-passing) model by setting *k*_on_ = *k*_off_ =0. NGF, nerve growth factor; p75, p75 neurotrophin receptor; TrkA, tropomyosin receptor kinase A.

We simulated the cross-membrane model to observe the dynamics of the ppErk signal upon stimulation with NGF in ligand-passing and heterodimer mechanisms for a broad range of *k*_on_ and *k*_off_ rates (see *Methods*). Initially, the model was simulated with no inhibitor present in the system (**Figure 2**). Under both mechanisms, the signal transiently rises, reaching a peak around 15–20 min after NGF stimulation and then eventually decreases and settles in a steady state; this behavior matches behavior of ppErk concentration observed in experiments upon NGF stimulation.^16^ The ppErk signal resulting from both mechanisms reached the same quantitative range. The ligand passing figure shows an overlap of several simulation trajectories, suggesting that the ligand passing model output is not sensitive to changes in the *k*_off_ parameter. However, overall, the simulations show no obvious differences between ligand passing and heterodimer mechanisms.

We next studied the effect of inhibitors on the signal transduction. The TrkA inhibitors were implemented in the model with the following reactions:

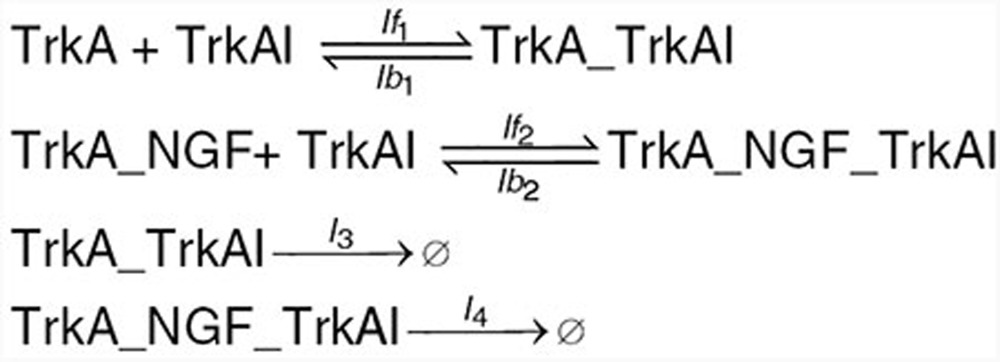


and the NGF inhibitor with the following reactions:

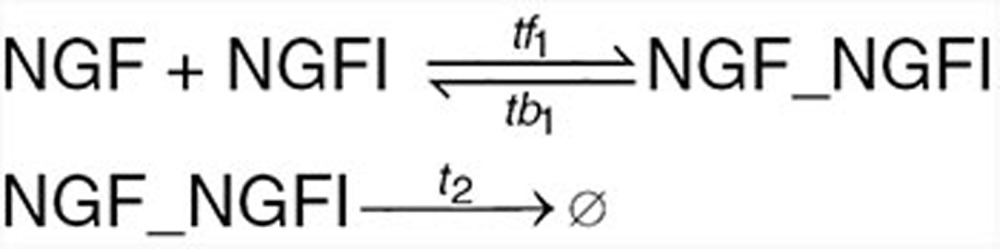


The simulations were first run until equilibrium before the inhibitors were added. NGF inhibitor was applied only once in the beginning of the simulation, while the TrkA inhibitor was applied twice a day (at times 0.5, 1, 1.5, 2, 2.5 days); this dosing schedule was chosen because the NGF inhibitor is longer lived in comparison to the TrkA inhibitor, which has a higher degradation rate^25^ (see parameter values in the **Supplementary Data**). Due to uncertainty in kinetic rates, simulations were performed for a broad range of rates (see *Methods*). The simulations are shown in **Supplementary Figures S1 and S2**. For a weak interaction between TrkA and p75 receptors (top right corner; see *Methods* for definition of “weak interaction”), the chosen values of the NGF inhibitor achieved better suppression of the signal than most of the TrkA inhibitor concentrations. For a strong interaction between TrkA and p75 (bottom left corner; see *Methods* for definition of “strong interaction”), the NGF inhibitor achieved significantly better suppression of the signal than all chosen concentrations of the TrkA inhibitor under the heterodimer mechanism (**Supplementary Figure S1**), while under ligand-passing mechanism, the suppression achieved with both inhibitors is comparable(**Supplementary Figure S2**). We note that upon stopping the treatment with the TrkA inhibitor, for some parameter combinations, the signal transiently increased to levels higher than the pretreatment steady-state level; this phenomena did not seem to be highly pronounced, and we did not investigate it further.

We noticed, however, that for the same simulations repeated with a different level of NGF stimulation, the conclusions about the effects of TrkA and NGF inhibitors did not necessarily agree with the above results.

### Dose prediction for the TrkA inhibitor

We next aimed to extend the results from the previous section to draw general conclusions about the performance of NGF and TrkA inhibitors and provide quantitative dose predictions to support drug discovery and development efforts for pain treatment.

Clinical trials have shown that 5 mg of tanezumab (i.e., NGF inhibitor) is efficacious in reducing pain.^24^ In order to guide the dose selection for the TrkA inhibitor, we used the model to predict the dose of the TrkA inhibitor that would result in the same effect as the NGF inhibitor, where the effect is measured by the nuclear ppErk concentration. We wanted to make this prediction independent of specific NGF concentration levels and independently of the *k*_on_ and *k*_off_ parameters of the p75–TrkA interaction, as these are unknown and/or variable (see *Methods* for definition of *k*_on_ and *k*_off_ parameters). Moreover, we were interested in whether the TrkA inhibitor dose can be predicted regardless of knowing the underlying p75–TrkA interaction mechanism.

**Figure 3:**
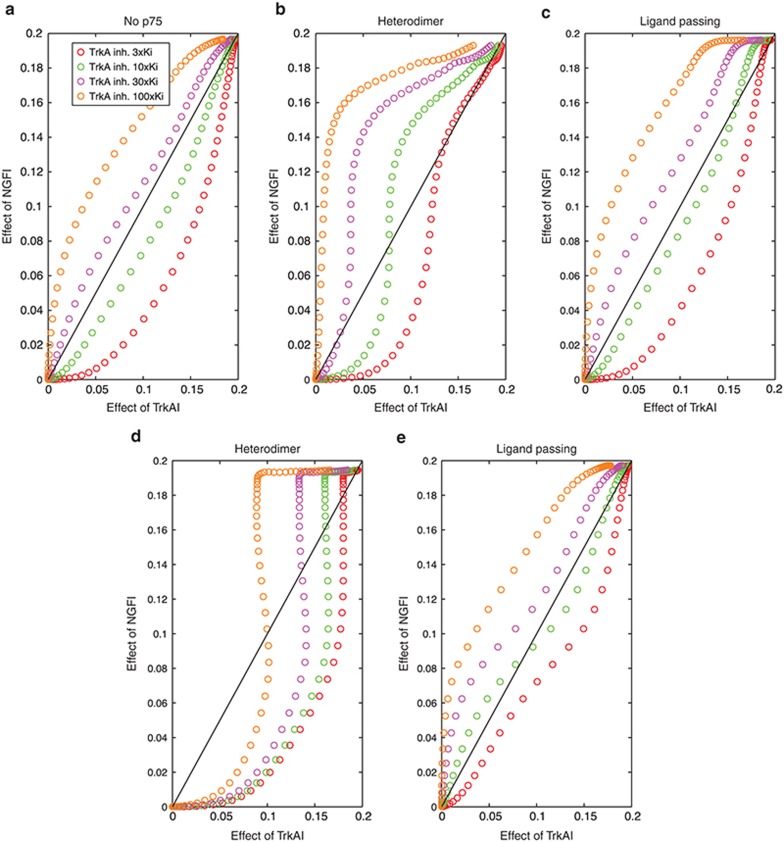
Comparison of effects of pathway inhibition with the NGF and TrkA inhibitors on the pathway output (dppErk). The effect (i.e., the minimum nuclear phosphorylated Erk concentration reached) of the NGF inhibitor (*x*-axis) is plotted against the effect of the TrkA inhibitor (*y*-axis). The NGF inhibitor was applied at 5 mg, and the TrkA inhibitor was applied at doses of 3K_i_ (red), 10K_i_ (green), 30K_i_ (magenta), and 100K_i_ (orange). Each circle represents the inhibitor effect for a different NGF synthesis rate and initial condition; synthesis rates were varied from 3.849 × 10^−11^ to 3.849 × 10^−4^ and initial concentration of NGF from 3 × 10^−8^ to 3 × 10^−1^. (**a**) No p75 in the system. (**b**) Heterodimer mechanism, medium p75–TrkA interaction strength. (**c**) Ligand-passing mechanism, medium p75–TrkA interaction strength. (**d**) Heterodimer mechanism, strong p75–TrkA interaction. (**e**) Ligand-passing mechanism, strong p75–TrkA interaction. NGF, nerve growth factor; p75, p75 neurotrophin receptor; TrkA, tropomyosin receptor kinase A.

**Figure 3** shows the effect achieved by inhibiting the NGF signaling pathway with the NGF inhibitor against the effect achieved by inhibiting the pathway with the TrkA inhibitor. The effect of inhibitors was evaluated by comparing the minimum points of pErk reached by the NGF inhibitor and TrkA inhibitor over the course of 3 days. The effect of 5 mg of NGF inhibitor was compared with 3Ki, 10Ki, 30Ki, and 100Ki of TrkA inhibitor. The NGF synthesis rate was varied across seven orders of magnitude (see *Methods*), and effects of inhibitors were calculated for each NGF synthesis rate. The effect of NGF inhibitor and TrkA inhibitor ranged from complete pErk repression (effect = 0 µM) to maximal pErk expression (effect = 0.2 µM). In cases when no p75 was present in the system (**Figure 3a**), or where p75–TrkA interaction was weak (low *k*_on_ and high *k*_off_ rates), NGF and TrkA inhibitor effect increased almost linearly for different NGF synthesis rates. The 5 mg dose of NGF inhibitor had an equivalent effect to the TrkA inhibitor dose between 10Ki and 30Ki. For medium strength of p75–TrkA interaction (**Figure 3b**,**c**), we observed similar linear relationship for the ligand-passing mechanism, where TrkA inhibitor dose between 10Ki and 30Ki was equivalent to 5 mg of NGF inhibitor (**Figure 3c**). However, for the heterodimer mechanism (**Figure 3b**), the relationship between TrkA inhibitor dose and the NGF inhibitor dose was more complex; for some NGF synthesis rates, treatment with NGF inhibitor was equivalent to 3Ki, while for the other values of NGF synthesis rate, it was equivalent to 30Ki and anything in between. For strong p75–TrkA interaction (high *k*_on_ and low *k*_off_ rates), ligand-passing mechanism started to deviate from linearity (**Figure 3e**), but we could nevertheless assume that TrkA inhibitor dose between 3Ki and 10Ki would give equivalent effect to 5 mg of NGF inhibitor. However, for the heterodimer mechanism (**Figure 3d**), the TrkA inhibitor dose heavily depended on the NGF synthesis rate. For some rates, high TrkA inhibitor dose such as 100Ki was not sufficient to match the effect of NGF inhibitor, while for others, the effect could be matched with less than 3Ki TrkA inhibitor dose.

In summary, we investigated what dose of TrkA inhibitor would match the effect of the 5 mg NGF inhibitor, and whether the answer depended on the (unknown) mechanism of the interaction between p75 and TrkA receptors. We found that for ligand-passing mechanism, 5 mg NGF inhibitor was equivalent to 10Ki to 30Ki TrkA inhibitor dose. For the heterodimer hypothesis, the relationship between the NGF inhibitor and TrkA inhibitor doses was nonlinear. For some NGF synthesis rates and p75–TrkA interaction parameters, TrkA inhibitor repressed pErk better than NGF inhibitor, while for others, the NGF inhibitor was significantly more successful in repressing pErk than the TrkA inhibitor. These results suggest that the understanding of the underlying p75–TrkA mechanism, and/or the values of NGF synthesis rate and *k*_on_/*k*_off_ kinetic rates, are crucial in predicting the TrkA inhibitor dose.

## Discussion

Identifying a drug target is a crucial milestone in early drug discovery. It takes an average of 15 years and huge financial investment to bring a new drug to the market, making it critical to select good targets carefully at the very beginning.^35^ Traditional approaches to drug discovery have been on a steep decline in delivering novel drugs in the past decades, despite committing increasing amounts of resources. Mistakes made in early stages of drug discovery often only become apparent in late stages during clinical trials, wasting valuable resources.

Modeling might be able to predict these unfortunate instances earlier. Modeling is comparatively inexpensive, and methods are becoming available for analyzing the dynamics of complex biological networks,^36^ for modeling biological variability and noise in molecular pathways,^37^ and for empirically inferring sources of patient variability in clinical responses.^38^ Modeling is also essential to deal with uncertainties such as incomplete knowledge of kinetic parameters and network topologies, allowing us to make robust conclusions about network interventions regardless of these uncertainties.^39^ To incorporate parameter uncertainty into our analysis, we have repeatedly performed simulations with varied parameter values; we mostly explored the effect of varying unknown complex binding and unbinding kinetic rates related to the new, p75-receptor part of the model, while keeping the previously published and experimentally fitted parameters fixed. Our approach thus corresponds to a form of local sensitivity analysis. A global or pseudo-global sensitivity analysis could in principle be performed to assess the effect of varying all the parameters. Novel tools for high-dimensional global sensitivity analysis have been a subject of ongoing research;^40,41^ however, for models with a high dimensional parameter space (our model has more than 250 parameters and 70 initial conditions), global sensitivity analysis remains challenging if not prohibitive due to high computational costs.

This manuscript makes a contribution to modeling the NGF signaling pathway, by including the p75 receptor into the model. We used the model to explore the implications of heterodimer and ligand-passing mechanisms for predicting the TrkA inhibitor dose for treating chronic pain. Under parameter uncertainty, we found that the TrkA inhibitor dose can be predicted for the ligand-passing mechanism, but not for the heterodimer mechanism. For the ligand-passing mechanism, we predicted the TrkA inhibitor dose that matches the working NGF inhibitor dose for treating chronic pain. For the heterodimer mechanism, though, knowledge of the NGF concentration as well as TrkA-p75 kinetic parameters is needed in order to be able to predict the TrkA inhibitor dose. These measurements are currently hard to perform due to technical limitations.

The above results point toward the need for better understanding of the p75–TrkA interaction. This inevitably leads to the question of what experiments to do to be able to distinguish between the ligand-passing and heterodimer mechanisms. Mechanistic modeling and studying the dynamics under different stimuli can give us insights into what experiment or a set of experiments will tell the models and therefore hypotheses apart.^42^

Adding the p75 receptor to the NGF signaling pathway model was one of the main contributions of this article. However, further improvements of the model are needed. For example, p75 has also been shown to promote retrograde transport^4,43^ and affect TrkA receptor endocytosis.^44,45^ Furthermore, activation of both TrkA and p75 receptors plays a major role in signaling through other pathways, such as PI3-kinase-protein kinase B (Akt) cascade;^46^ however, in this article, we only focused on the MAP kinase pathway signaling pathway branch. It would be interesting to study the effect that NGF and TrkA inhibitors have on other pathways. Such analysis could contribute to understanding side effects of pain treatment drugs. We also note that other hypotheses have been proposed as explanations for the p75–TrkA interactions, besides the ligand-passing and heterodimer hypotheses. For example, proteins such as ankyrin-rich membrane spanning protein, which is often coexpressed with TrkA and p75, might interact with both receptors and acts as a scaffolding protein that promotes and stabilizes the interaction. Another possible mechanism that could explain alterations in NGF affinity is clustering of receptors in lipid rafts.^47^ Similarly, as in this article, mathematical models could be developed and studied for those hypotheses. Another area that needs further investigation is the relationship between dppErk and pain.^25^ Phosphorylated Erk has been known to trigger expression of numerous genes related to neuronal survival and pain sensation. However, the precise quantitative relationship between dppErk and pain outcomes remain to be investigated further.

Systems pharmacology has only just started making contributions to drug discovery and development. Ultimately, the questions that systems pharmacology can help us answer are much broader that what we aimed for in this manuscript: Are there better/worse drug targets in a pathway? What is an optimal combination therapy? And how can understanding patient variability improve treatments?

## Methods

All models were developed and analyzed using MATLAB/SIMBIOLOGY version 2012b or 2013a (MATLAB, Natick, MA). Cross membrane molecular interactions were modeled using the approach of Benson *et al*.^25^ Kinetic rates of NGF binding and unbinding from p75 and TrkA receptors were obtained from Mahadeo *et al.*^31^ (**Table 1**). The rates *l*2 and *h*4 and of a breakup of a complex TrkA_NGF_p75 (and TrkA_NGF_p75_d_ in a heterodimer mechanism) into TrkA_NGF and p75 was chosen as 100 s^−1^ as the complex has been known to be extremely short lived.^15^ For the rates of which values are unknown, we have performed simulations for a number of parameter combinations. Specifically, association and dissociation rates of complex formation of TrkA and p75 in the heterodimer hypothesis, and association and dissociation rates of TrkA binding to NGF–p75 complex, were chosen as *k*_on_ = {0.005, 0.01, 0.05, 0.1, 1} M^−1^·s^−1^ and *k*_off_ = 6.4 × {10^−5^, 10^−4^, 10^−3^, 10^−2^, 10^−1^, 1} s^−1^. The initial concentrations of p75 were chosen as 10 times the initial concentrations of TrkA.^48,49^ Prior to introducing inhibitors, all species were initialized to their equilibrium values. Binding and degradation rates for the NGF inhibitor were chosen to mimic those of the tanezumab^25,50^ and rates for TrkA inhibitor were chosen to match those used in ref. 34 and *K*_i_ = 1.18 × 10^−3^ nM. When referring to *k*_on_ and *k*_off_ rates of the p75–TrkA interaction, we specifically refer to *hf*_1_ and *hb*_1_ and for the heterodimer mechanism and *lf*_1_ and *lb*_1_ and for the ligand-passing mechanism. Expressions weak, medium, and strong p75–TrkA interaction strength are used as follows: for strong interaction, we use high *k*_on_ (*hf*_1_ and *lf*_1_) rates and low *k*_off_ (*hb*_1_ and *lb*_1_) rates; for weak interaction, low *k*_on_ rates and high *k*_off_ rates; for medium interaction, we use rates values that fall between the strong and week values. NGF production rate *if*_1_ was varied from 3.849 × 10^−11^ to 3.849 × 10^−4^ and initial concentration of NGF from 3 × 10^−8^ to 3 × 10^−1^. This range of NGF synthesis rates and concentrations were selected based on concentrations reported in the experimental literature which span a broad range from pmol/l to high nmol/l.^16,25^ Physiological parameters were incorporated into the cross-compartmental model, for which we used the volumes of 15l for the interstitial fluid compartment (extracellular body water compartment) and 0.001l for the neuron. Unless otherwise specified, all other parameter values and initial conditions are the same as in ref. 25 or ref. 34.

## Author Contributions

T.T., P.D., and P.H.v.d.G conceived and designed the experiments. T.T. performed the experiments. T.T. analyzed the data. T.T., P.D., and P.H.v.d.G. wrote the manuscript. Editor-in-Chief P.H.v.d.G. was not involved in the *CPT:PSP* review or decision process for this article.

## Conflict of Interest

The authors declared no conflict of interest. As Editor-in-Chief of CPT:PSP, P.H v.d.G was not involved in the review or decision process for this paper.

## Study Highlights


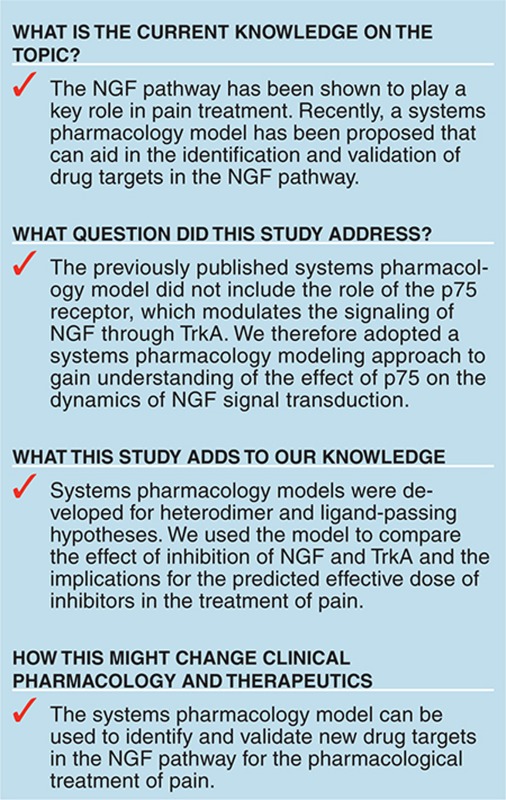


## Figures and Tables

**Figure 1:**
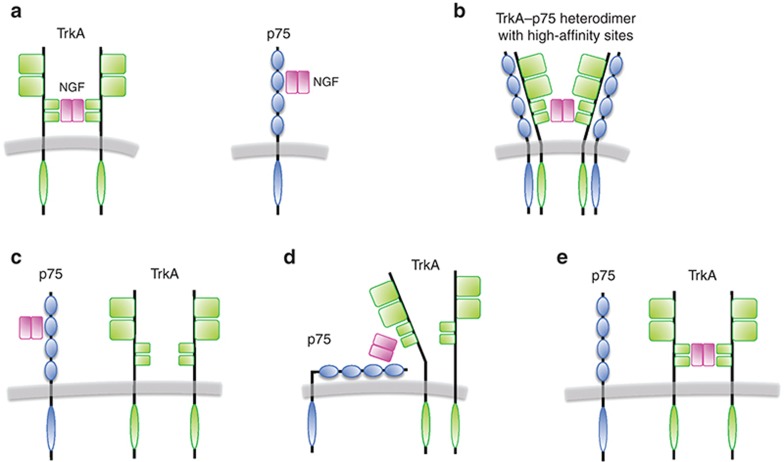
Schematic representation of the ligand-passing and heterodimer mechanisms. (**a**) Dimeric NGF ligand binds the p75 monomer and the TrkA dimer. (**b**) The heterodimer mechanism. (**c–e**) The ligand-passing mechanism. NGF, nerve growth factor; p75, p75 neurotrophin receptor; TrkA, tropomyosin receptor kinase A.

**Table 1:**
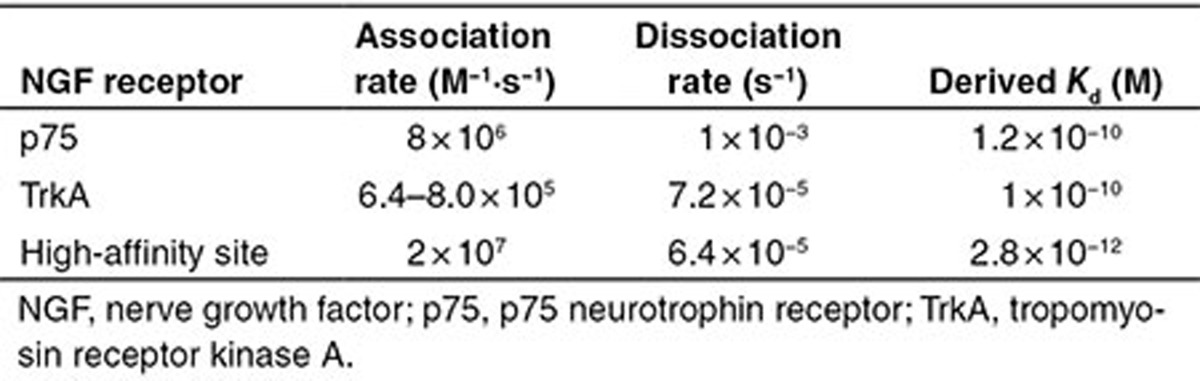
Kinetic rates of NGF binding TrkA and p75 receptors^31^
