# Supplementary material for: Systems Pharmacology of the NGF Signaling Through p75 and TrkA Receptors
Source: CPT Pharmacometrics Syst Pharmacol. 2014 Dec 3;3(12):e150–. doi: 10.1038/psp.2014.48 (PMC4288001; doi:10.1038/psp.2014.48)
Supplement: Supplementary Information [file psp201448x1.zip › PSP-2014-0062-s04.pdf]

Additional file 3: Systems pharmacology of the NGF signalling through p75 and TrkA receptors

Tina Toni, Pinky Dua, Piet van der Graaf

The simple model

Common reactions

$$
\begin{aligned}
\emptyset &\xrightarrow{kf_1} NGF \xrightarrow{kb_1} \emptyset \\
\text{proTrkA} &\xrightarrow{kf_2} \text{TrkA} \xrightarrow{kb_2} \emptyset \\
\text{prop75} &\xrightarrow{kf_3} \text{p75} \xrightarrow{kb_3} \emptyset \\
\text{TrkA} + \text{NGF} &\underset{kb_4}{\overset{kf_4}{\rightleftharpoons}} \text{TrkA\_NGF} \\
\text{p75} + \text{NGF} &\underset{kb_5}{\overset{kf_5}{\rightleftharpoons}} \text{p75\_NGF} \\
\text{TrkA\_NGF} &\xrightarrow{kf_6} \text{pTrkA}
\end{aligned}
$$

Heterodimer model

$$
\begin{aligned}
\text{TrkA} + 2\text{p75} &\underset{hb_1}{\overset{hf_1}{\rightleftharpoons}} \text{TrkA\_p75}_\text{d} \\
\text{TrkA\_p75}_\text{d} &\xrightarrow{h_2} \emptyset \\
\text{NGF} + \text{TrkA\_p75}_\text{d} &\underset{hb_3}{\overset{hf_3}{\rightleftharpoons}} \text{TrkA\_NGF\_p75}_\text{d} \\
\text{TrkA\_NGF\_p75}_\text{d} &\xrightarrow{h_4} \text{TrkA\_NGF} + 2\text{p75}
\end{aligned}
$$

Ligand-passing model

$$
\begin{aligned}
\text{TrkA} + \text{p75\_NGF} &\underset{lb_1}{\overset{lf_1}{\rightleftharpoons}} \text{TrkA\_NGF\_p75} \\
\text{TrkA\_NGF\_p75} &\xrightarrow{l_2} \text{TrkA\_NGF} + \text{p75}
\end{aligned}
$$

TrkA inhibitor reactions

$$\mathrm{TrkA} + \mathrm{TrkAI} \underset{Ib_1}{\overset{If_1}{\rightleftharpoons}} \mathrm{TrkA\_TrkAI}$$

$$\mathrm{TrkA\_NGF} + \mathrm{TrkAI} \underset{Ib_2}{\overset{If_2}{\rightleftharpoons}} \mathrm{TrkA\_NGF\_TrkAI}$$

$$\mathrm{TrkA\_TrkAI} \xrightarrow{I3} \emptyset$$

$$\mathrm{TrkA\_NGF\_TrkAI} \xrightarrow{I4} \emptyset$$

NGF inhibitor reactions

$$\mathrm{NGF} + \mathrm{NGFI} \underset{tb_1}{\overset{tf_1}{\rightleftharpoons}} \mathrm{NGF\_NGFI}$$

$$\mathrm{NGF\_NGFI} \xrightarrow{t_2} \emptyset$$

Table 1: Parameter values

parameter	value	unit
kf_1	4.25E-8	min^{-1}
kb_1	0.001283	min^{-1}
kf_2	8.3333E-4	s^{-1}
kb_2	2.7833E-4	s^{-1}
kf_3	same as kf_2	
kb_3	same as kb_2	
kf_4	0.8	$micromolarity^{-1}s^{-1}$
kb_4	7.2E-5	s^{-1}
kf_5	8.0	$micromolarity^{-1}s^{-1}$
kb_5	0.001	s^{-1}
k_6	1.0	s^{-1}
hf_1	6.2	$micromolarity^{-2}s^{-1}$
hb_1	6.4E-5	s^{-1}
h_2	2.7833E-4	s^{-1}
hf_3	20.0	$micromolarity^{-1}s^{-1}$
hb_3	6.4E-5	s^{-1}
h_4	100	s^{-1}
lf_1	6.2	$micromolarity^{-1}s^{-1}$
lb_1	6.4E-5	s^{-1}
l_2	100	s^{-1}

Table 2: Initial conditions

species name	value	unit
TrkA	0.06189368	micromolarity
p75	0.6189368	micromolarity
NGF	3.0E-5	micromolarity
TrkA_NGF	0.0	micromolarity
p75_NGF	0.0	micromolarity
pTrkA	0.0	micromolarity
TrkA_NGF_p75	0.0	micromolarity
TrkA_NGF_$p75_d$	0.0	micromolarity
proTrkA	0.020631	micromolarity
prop75	0.20631	micromolarity
source	1.0	micromolarity
