# Supplementary material for: Systems Pharmacology of the NGF Signaling Through p75 and TrkA Receptors
Source: CPT Pharmacometrics Syst Pharmacol. 2014 Dec 3;3(12):e150–. doi: 10.1038/psp.2014.48 (PMC4288001; doi:10.1038/psp.2014.48)
Supplement: Supplementary Information [file psp201448x1.zip › PSP-2014-0062-s05.pdf]

Additional file 4: Systems pharmacology of the NGF signalling through p75 and TrkA receptors

Tina Toni, Pinky Dua, Piet van der Graaf

The cross-membrane model

Cross membrane

$$\text{[int.flu]TrkA\_int\_flu} \underset{cb_1}{\overset{cf_1}{\rightleftharpoons}} \text{Neuron.TrkA}$$

$$\text{[int.flu]p75\_int\_flu} \underset{cb_2}{\overset{cf_2}{\rightleftharpoons}} \text{Neuron.p75}$$

$$\text{[int.flu]TrkA\_NGF\_int\_flu} \underset{cb_3}{\overset{cf_3}{\rightleftharpoons}} \text{Neuron.TrkA\_NGF}$$

$$\text{[int.flu]p75\_NGF\_int\_flu} \underset{cb_4}{\overset{cf_4}{\rightleftharpoons}} \text{Neuron.p75\_NGF}$$

$$\text{[int.flu]TrkA\_p75}_{\text{d}}\text{\_int\_flu} \underset{cb_5}{\overset{cf_5}{\rightleftharpoons}} \text{Neuron.TrkA\_p75}_{\text{d}} \quad \text{(Heterodimer)}$$

$$\text{[int.flu]TrkA\_NGF\_p75}_{\text{d}}\text{\_int\_flu} \underset{cb_6}{\overset{cf_6}{\rightleftharpoons}} \text{Neuron.TrkA\_NGF\_p75}_{\text{d}} \quad \text{(Heterodimer)}$$

$$\text{[int.flu]TrkA\_NGF\_p75\_int\_flu} \underset{cb_7}{\overset{cf_7}{\rightleftharpoons}} \text{Neuron.TrkA\_NGF\_p75} \quad \text{(Ligand-passing)}$$

Neuron

$$\text{Neuron.pro\_TrkA} \xrightarrow{nf1} \text{Neuron.TrkA} \xrightarrow{nb_1} \emptyset$$

$$\text{Neuron.pro\_p75} \xrightarrow{nf2} \text{Neuron.p75} \xrightarrow{nb_2} \emptyset$$

$$\text{Neuron.TrkA\_NGF} \xrightarrow{n_3} \text{Neuron.pTrkA}$$

$$\text{Neuron.TrkA} + 2\text{Neuron.p75} \underset{nb_4}{\overset{nf_4}{\rightleftharpoons}} \text{Neuron.TrkA\_p75}_{\text{d}} \quad \text{(Heterodimer)}$$

$$\text{Neuron.TrkA\_p75}_{\text{d}} \xrightarrow{n_5} \emptyset \quad \text{(Heterodimer)}$$

$$\text{Neuron.TrkA\_NGF\_p75}_{\text{d}} \xrightarrow{n_6} \text{Neuron.TrkA\_NGF} + 2\text{Neuron.p75} \quad \text{(Heterodimer)}$$

$$\text{Neuron.TrkA} + \text{Neuron.p75\_NGF} \underset{nb_7}{\overset{nf_7}{\rightleftharpoons}} \text{Neuron.TrkA\_NGF\_p75} \quad \text{(Ligand-passing)}$$

$$\text{Neuron.TrkA\_NGF\_p75} \xrightarrow{n_8} \text{Neuron.TrkA\_NGF} + \text{Neuron.p75} \quad \text{(Ligand-passing)}$$

Interstitial fluid

$$[\text{int.flu}]\text{source} \xrightarrow{if_1} [\text{int.flu}]\text{NGF} \xrightarrow{ib_1} [\text{int.flu}]\text{NGFdeg}$$

$$[\text{int.flu}]\text{TrkA\_int\_flu} + [\text{int.flu}]\text{NGF} \underset{ib_2}{\overset{if_2}{\rightleftharpoons}} [\text{int.flu}]\text{TrkA\_NGF\_int\_flu}$$

$$[\text{int.flu}]\text{p75\_int\_flu} + [\text{int.flu}]\text{NGF} \underset{ib_3}{\overset{if_3}{\rightleftharpoons}} [\text{int.flu}]\text{p75\_NGF\_int\_flu}$$

$$[\text{int.flu}]\text{TrkA\_p75}_\text{d}\text{\_int\_flu} + [\text{int.flu}]\text{NGF} \underset{ib_4}{\overset{if_4}{\rightleftharpoons}} [\text{int.flu}]\text{TrkA\_NGF\_p75}_\text{d}\text{\_int\_flu (Heterodimer)}$$

TrkA inhibitor reactions

$$[\text{int.flu}]\text{TrkAI\_int\_flu} \xrightarrow{i5} \emptyset$$

$$[\text{int.flu}]\text{TrkAI\_int\_flu} \underset{cb_8}{\overset{cf_8}{\rightleftharpoons}} \text{Neuron.TrkAI}$$

$$\text{Neuron.TrkA} + \text{Neuron.TrkAI} \underset{Ib_1}{\overset{If_1}{\rightleftharpoons}} \text{Neuron.TrkA\_TrkAI}$$

$$\text{Neuron.TrkA\_NGF} + \text{Neuron.TrkAI} \underset{Ib_2}{\overset{If_2}{\rightleftharpoons}} \text{Neuron.TrkA\_NGF\_TrkAI}$$

$$\text{Neuron.TrkA\_TrkAI} \xrightarrow{I3} \emptyset$$

$$\text{Neuron.TrkA\_NGF\_TrkAI} \xrightarrow{I4} \emptyset$$

NGF inhibitor reactions

$$[\text{int.flu}]\text{NGFI} \xrightarrow{i6} \emptyset$$

$$[\text{int.flu}]\text{NGF} + [\text{int.flu}]\text{NGFI} \underset{tb1}{\overset{tf1}{\rightleftharpoons}} [\text{int.flu}]\text{NGF\_NGFI}$$

$$[\text{int.flu}]\text{NGF\_NGFI} \xrightarrow{t2} \emptyset$$

Table 1: Parameter values

parameter	value	unit
nf_1	0.049998	min^{-1}
nb_1	0.0166668	min^{-1}
nf_2	same as nf1	
nb_2	same as nb1	
n_3	60.0	min^{-1}
nf_4	372.0	$micromolarity^{-2} min^{-1}$
nb_4	0.00384	min^{-1}
n_5	0.0166668	min^{-1}
n_6	6000.0	min^{-1}
nf_7	372.0	$micromolarity^{-1} min^{-1}$
nb_7	0.00384	min^{-1}
n_8	6000.0	min^{-1}
if_1	3.849E-8	min^{-1}
ib_1	0.001283	min^{-1}
if_2	48.0	$micromolarity^{-1} min^{-1}$
ib_2	0.0043	min^{-1}
if_3	480.0	$micromolarity^{-1} min^{-1}$
ib_3	0.06	min^{-1}
if_4	1200.0	$micromolarity^{-1} min^{-1}$
ib_4	0.00384	min^{-1}
cf_1	2000.0	min^{-1}
cb_1	2000.0	min^{-1}
cf_2	same as cf1	
cb_2	same as cb1	
cf_3	same as cf1	
cb_3	same as cb1	
cf_4	same as cf1	
cb_4	same as cb1	
cf_5	same as cf1	
cb_5	same as cb1	
cf_6	same as cf1	
cb_6	same as cb1	
cf_7	same as cf1	
cb_7	same as cb1	
i_5	0.001925	min^{-1}
cf_8	83.33	min^{-1}
cb_8	1000000.0	min^{-1}
If_1	1.0E7	$molarity^{-1} s^{-1}$
Ib_1	0.0118	s^{-1}
If_2	same as If1	
Ib_2	same as Ib1	
I_3	0.0167	min^{-1}
I_4	same as I3	
i_6	2.3E-5	min^{-1}
tf_1	16.2	$micromolarity^{-1} min^{-1}$
tb_1	1.8E-4	min^{-1}
t_2	2.3E-5	min^{-1}

Table 2: Initial conditions

compartment	species name	value	
Neuron	TrkA	0.0606	micromolarity
Neuron	TrkA_NGF	0.1819	micromolarity
Neuron	pTrkA	0.0	micromolarity
Neuron	TrkAI	0.0	micromolarity
Neuron	pro_TrkA	0.020631	micromolarity
Neuron	TrkA_NGF_TrkAI	0.0	micromolarity
Neuron	TrkA_TrkAI	0.0	micromolarity
Neuron	p75	0.606	micromolarity
Neuron	p75_NGF	1.819	micromolarity
Neuron	TrkA_NGF_p75	0.0	micromolarity
Neuron	TrkA_NGF_$p75_{d}$	0.0	micromolarity
Neuron	TrkA_$p75_{d}$	0.0	micromolarity
Neuron	pro_p75	0.20631	micromolarity
interstitial fluid	TrkA_int_flu	5.0E-6	micromolarity
interstitial fluid	source	1.0	micromolarity
interstitial fluid	NGF	3.0E-5	micromolarity
interstitial fluid	TrkA_NGF_int_flu	1.515E-5	micromolarity
interstitial fluid	NGFI	0.0	micromolarity
interstitial fluid	NGF_NGFI	0.0	micromolarity
interstitial fluid	TrkAI_int_flu	0.0	micromolarity
interstitial fluid	p75_int_flu	5.0E-5	micromolarity
interstitial fluid	p75_NGF_int_flu	1.515E-4	micromolarity
interstitial fluid	TrkA_NGF_p75_int_flu	0.0	micromolarity
interstitial fluid	TrkA_NGF_$p75_{d}$_int_flu	0.0	micromolarity
interstitial fluid	TrkA_$p75_{d}$_int_flu	0.0	micromolarity
