# Supplementary figures and images for: Systems Pharmacology of the NGF Signaling Through p75 and TrkA Receptors
Source: CPT Pharmacometrics Syst Pharmacol. 2014 Dec 3;3(12):e150–. doi: 10.1038/psp.2014.48 (PMC4288001; doi:10.1038/psp.2014.48)

# Heterodimer

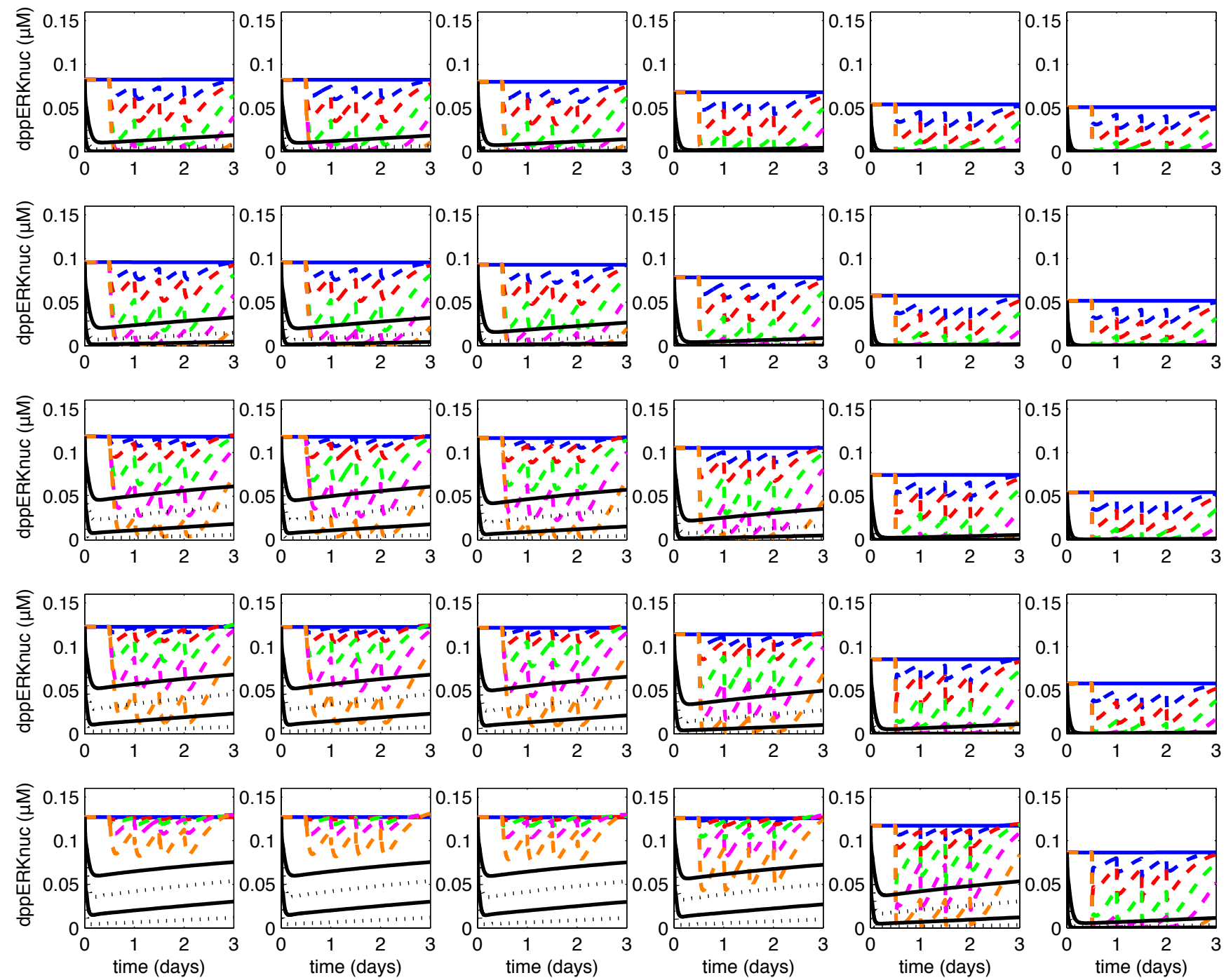

Supplement: Supplementary Information [file psp201448x1.zip › PSP-2014-0062-s02.pdf]

Ligand passing

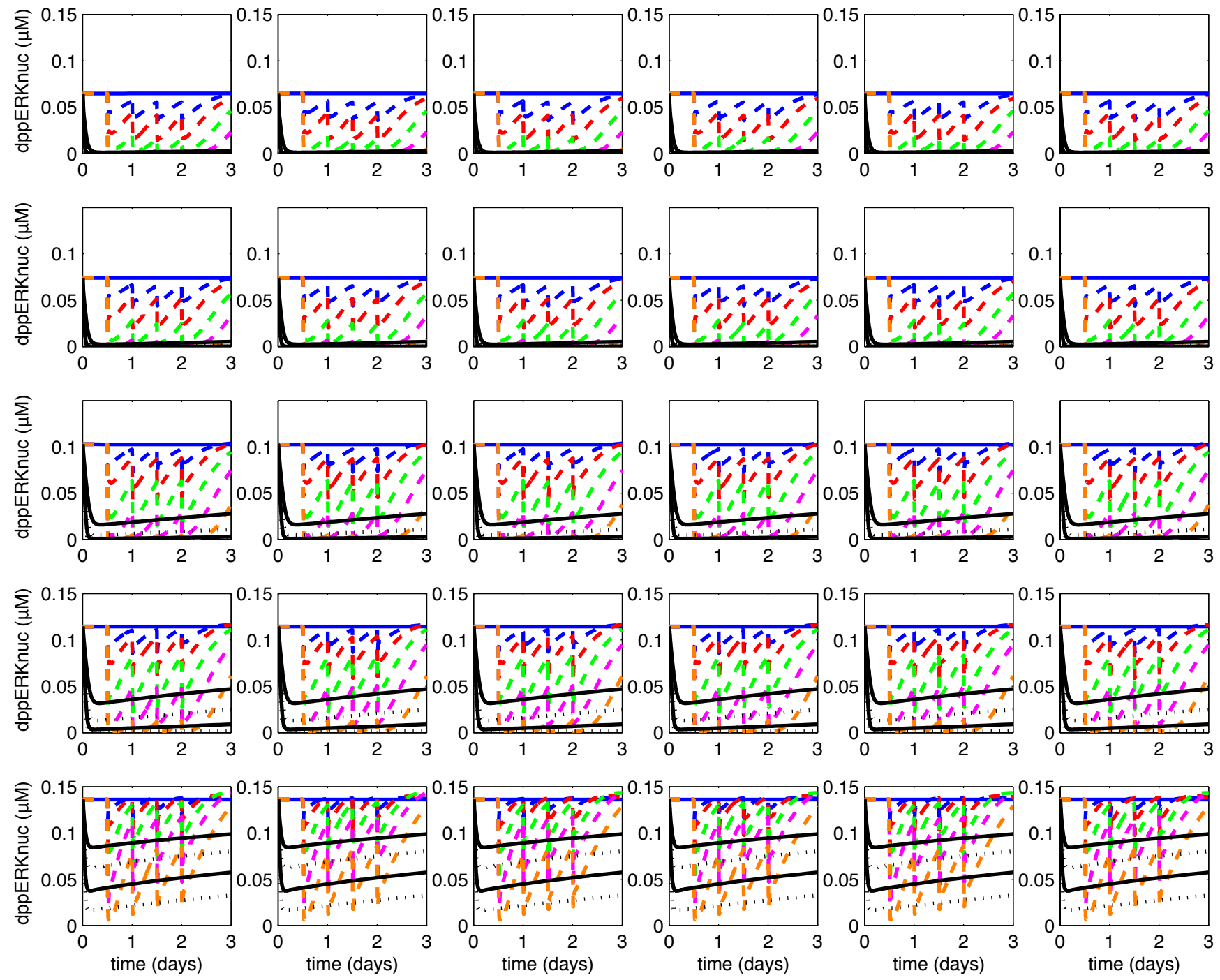

Supplement: Supplementary Information [file psp201448x1.zip › PSP-2014-0062-s03.pdf]
